# Deep learning-assisted tools to understand the structural biology of the synapse

**DOI:** 10.1007/s13534-025-00512-5

**Published:** 2025-10-21

**Authors:** Zoltán Gáspári, Zsófia E. Kálmán, Anna Sánta

**Affiliations:** Faculty of Information Technology and Bionics, Pázmány Péter Catholic University, Práter u. 50/A, Budapest, 1083 Hungary

**Keywords:** Postsynaptic density, Multivalent proteins, Protein structure prediction, Protein complex modeling

## Abstract

The function of our brain is the result of the balanced interplay between billions of neurons forming a network of enormous complexity. However, the neurons themselves are also immensely complex entities, with many specialized macromolecular structures orchestrating signal processing and propagation. The postsynaptic density is an elaborate network of interconnected proteins, a dynamic yet highly organized molecular assembly beneath the dendritic membrane, and plays a pivotal role in learning, memory formation, and the development of a number of cognitive disorders. In this review, we argue that with the recent blooming of AI-assisted computational tools in structural biology, we might be able to get closer to understanding the molecular-level mechanistic aspects of this machinery. Nevertheless, we have to use these methods with caution as they are not yet capable of solving all the questions that arise for such a complex macromolecular system. First, we focus on the unique features of the postsynaptic protein network, highlighting those that pose particular challenges for such a modeling task, and put these in the light of the currently available deep learning-based approaches. We highlight the aspects that need specific attention and the areas where future developments could facilitate the detailed description of neural function at the molecular level.

## Introduction

Understanding how the human brain works is one of the biggest, if not the single largest challenge of biology [1]. The immense complexity of the network formed by billions of neurons [2] seems an insurmountable problem which can only be studied by systematic analysis of smaller systems and/or focusing on highly specific questions. The picture is further complicated by the processes at the cellular level, where neurons pose additional difficulties as they have a number of unique features compared to other kinds of cells [3]. Moreover, there are numerous different neuron types differing in many morphological and functional aspects [4]. However, linking genetic conditions to phenotype, in this case, the observed behavioral and cognitive features of individuals, requires that we have at least a qualitative explanation of the underlying mechanisms of how the various molecules and their interplay contribute to the functions of individual neurons, and how these influence information processing by larger networks.

Deep learning-based approaches are currently skyrocketing in practically all areas of life and science, structural biology being no exception. The flagship method in this field is AlphaFold, and its impact is clearly reflected by awarding the 2024 Nobel prize in Chemistry to its developers [5]. AlphaFold and related methods indeed open novel possibilities in protein structure research, and are expected to provide access to model and understand systems that are hard or costly to explore with current experimental techniques. The performance of complex, deep learning-based approaches often surpasses that of more traditional computational methods. Bright as the future of AI-assisted structural biology might be, as with each methodology, we should use it and treat the results with healthy caution, and we should be aware of the potential pitfalls [6]. It is nevertheless inevitable that future developments will bring even more tools for the investigation of increasingly complex problems. In this review, we attempt to enumerate some of the major challenges in the structural modeling of molecules and multimers important in neural complexes. In our view, due to its complexity, this area integrates a number of aspects currently at the frontline of methodological developments and even beyond, thus, the postsynaptic protein network provides a unique demonstrative case to list and evaluate some of the state-of-the-art methodology. Below we provide a brief overview on some of the currently available modeling tools with special focus on recent AI-based developments. Finally, we identify some of the problems and aspects that could be addressed in the future for more effective description of neural function at the molecular level. We argue that innovative integration of the rapidly developing experimental techniques in neurobiology with advanced deep learning-based computational approaches could have the potential to provide long-sought insights on the molecular mechanisms and their contribution to function at a larger scale.

## Understanding molecular aspects of human cognition

Neurons are specialized cells that receive, process, and transmit information, most often, but not exclusively, to and from other neurons. The most well-known unique functional aspect of neurons is their ability to transmit electrical signals along their cell membrane. The basic mechanisms of initiating and transmitting such signals, like the propagation of the action potential, are well understood along with the main principles of neuron-neuron communication [3]. The main form of interneural signal transmission is the chemical synapse, most often formed between the axon of one cell and a dendritic spine of another. The synapse is composed of a presynaptic part, where neurotransmitters are released, a postsynaptic part, capable of detecting these molecules, and the synaptic cleft in between. In excitatory synapses, release of neurotransmitter molecules like glutamate cause the postsynaptic membrane to generate an action potential that will travel along the cell membrane. Inhibitory synapses act against action potential initiation or propagation. A single neuron can have thousands of synapses from which it gets incoming signals but is generally believed to produce a single output – after integrating the incoming signals, it will either “fire” or not.

The complexity of neural functions is usually considered to arise from the network architecture of the nervous system, i.e. the actual interconnections between neurons and the dynamic reorganization of these [7]. However, single-cell transcriptomic studies made it clear that neurons have not only different morphologies but might possess an even higher diversity in their biomolecular composition [8]. Neurons seem to possess a specific molecular machinery that plays a key role in the modulation of these signals and in orchestrating different kinds of cellular responses [9]. The postsynaptic density, an elaborate protein network beneath the postsynaptic membrane, is a prime example of such a complex molecular machinery.

The postsynaptic density (PSD) obtained its name from its appearance in electron microscopy images where it is observed as a dark structure beneath the postsynaptic membrane, most pronounced in excitatory synapses. It is actually a highly interconnected network of different proteins, linking transmembrane neurotransmitter receptors to the cytoskeletal elements, most prominently actin filaments, that play a key role in the formation of the dendritic spines. The PSD is believed to be highly important in the molecular-level processes of learning and memory, and mutations in a number of PSD proteins have been suggested to be associated with altered cognitive functions [10, 11]. In particular, certain forms of intellectual disability, autism spectrum disorder (ASD), and schizophrenia are among the conditions linked to changes in PSD functions. The composition of the PSD is variable among neuron types, and this prompted the formulation of the “synaptomic theory” that emphasizes the differences between the complex molecular composition of synapses as a major contributor to neuron function, as opposed to the paradigm relying on the exclusive role of the architecture of neural networks [12].

The postsynaptic density has not only different protein composition in various cells but can also dynamically change its internal structure. The PSD is restructuring itself during sleep, in which the availability of specific protein isoforms (variants) is key. The PSD also changes its size upon stimuli and exhibits both immediate and long-term changes as a response to neural activity [13, 14]. While these rearrangements are often accompanied by the change of the amount of individual protein components, it is also believed that some restructuring can take place even when the protein set is relatively constant. Protein composition changes can occur via local protein synthesis, typically from locally stored mRNA pools, and postsynaptic proteins act as regulators of this process [15].

**Fig. 1:**
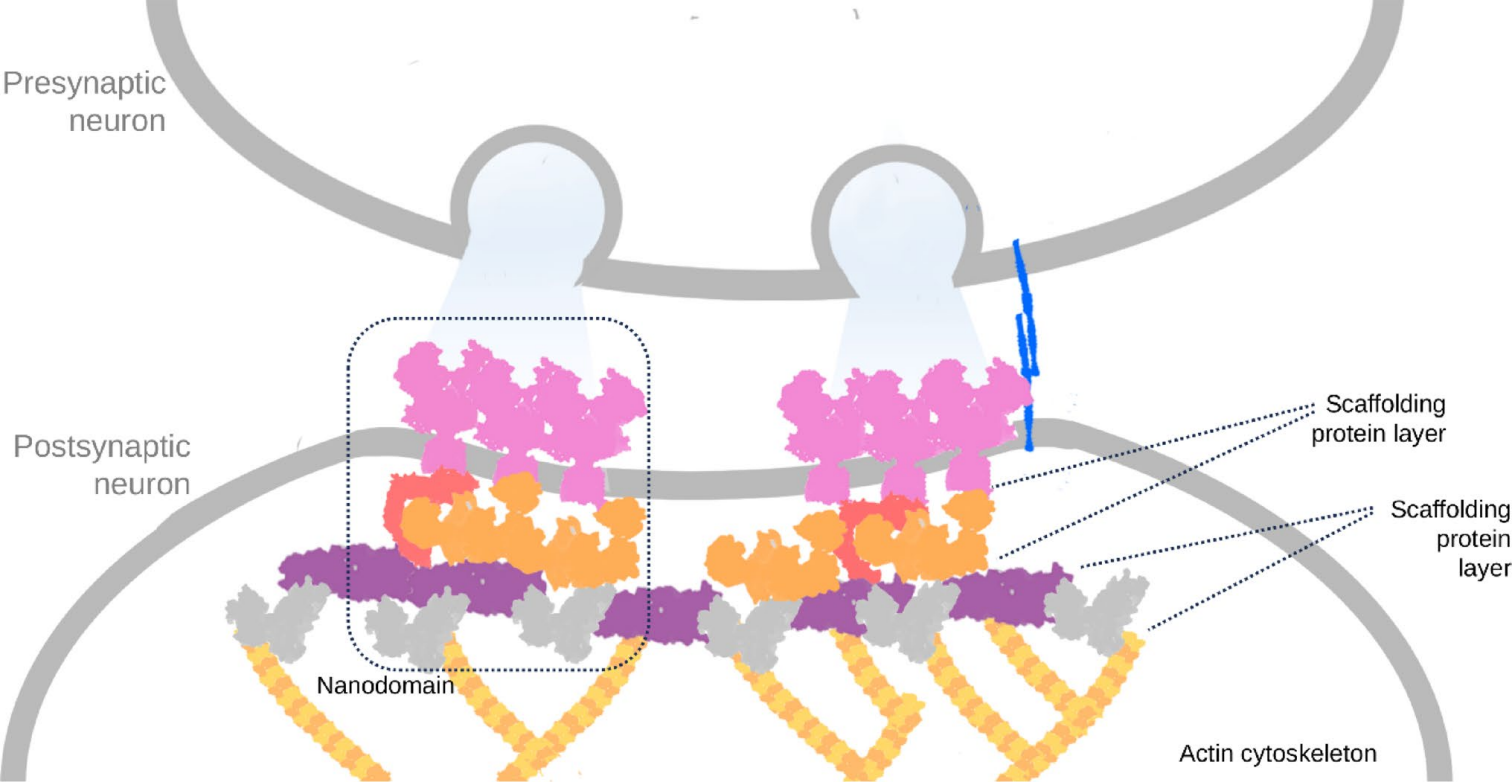
Schematic architecture of the postsynaptic density, emphasising both its layered organization and the presence of functional nanodomains, depicted to align with presynaptic active zones of neurotransmitter release

The dynamic aspect does not mean that the PSD is devoid of an internal structure. It is actually a highly organized protein network in three dimensions [16]. It exhibits a layered architecture, where the proteins directly associating with transmembrane receptors are linked to a different set of molecules towards the inside of the cell, which are further linked to the cytoskeleton. It also forms so-called nanodomains, functional units perpendicular to the membrane that group receptors into defined regions of the postsynaptic membrane (Fig. 1). The number and size of nanodomains can vary, and there is growing evidence that the nanodomains are aligned with presynaptic structures for efficient signal transduction [17, 18].

It is clear that a detailed description of the structural organization of the PSD would be needed to account for its functional role and to understand the pathogenesis of intellectual disorders in sufficient detail. However, deciphering the molecular-level organization of the PSD is one of the most difficult problems in structural biology today. Below we summarize some of the most challenging aspects, and will reflect on these in the light of the most recent breakthroughs in structural biology methods utilizing neural networks.

### Presence of different protein structural classes

The postsynapse contains almost all kinds of proteins known to date [19]. The receptors binding the neurotransmitters are large transmembrane proteins, often multisubunit complexes like the NMDA and AMPA receptors. Beneath the membrane, there are long scaffold proteins [20] that typically exhibit extended intrinsically disordered segments. There are also fibrillar segments like coiled coils and single alpha-helices. From the cytoplasmic side, there are actin filaments formed by monomers that dynamically polymerize and depolymerize. Of course, there are also many protein segments that belong to the best-known globular class, which, as will be elaborated below, are the proteins that are best characterized and easiest to model using the current structural bioinformatics toolkit. This kind of structural diversity is currently very hard to tackle, as will be detailed below.

### Non-uniform stoichiometry and multivalency

As described above, the PSD does not have a fixed composition neither in terms of the types of proteins nor in their ratio relative to each other [21]. In addition, the PSD is like a set of Lego bricks: from the same set, many different structures can be assembled. The major reason for this is that many long scaffold proteins are multivalent, meaning that they have multiple sites where they can interact with the same partner. Some proteins have more than one partner for which they have two or more binding sites, making the number of theoretically possible assemblies especially large [22]. Add the changes in protein composition to the mix and then we have very few fixed points to rely on when trying to build models. A key question is the presence of structural and functional constraints that restrict and regulate the composition and architecture of the assemblies actually occurring *in vivo.*

### Dynamic nature and phase separation

Closely related and not separable from its variability, the PSD is dynamic as it can rearrange both upon the addition and extraction of components. It is also suggested that the PSD can change its structure even while largely retaining its protein composition. In this respect, the presence of flexible, intrinsically disordered segments, as mentioned above, can be of utmost importance. One possible explanation for this dynamic aspect could be the presence of protein condensates [23]. These assemblies are generated by liquid–liquid phase separation, a relatively recently discovered phenomenon that is increasingly recognized to play an important role in many biomolecular processes. Condensates might act as protein reservoirs to store unused proteins but also as sites of active biochemical events. Condensates can be generated and dissolved by specific protein modifications such as phosphorylation but also upon incoming electrochemical stimuli, mediated by small changes in the pH at the postsynapse [24].

## Use of deep learning-based methods in the structural biology of synaptic proteins

It is likely impossible to provide a comprehensive summary of deep learning-based tools in structural biology because of the breathtaking pace at which novel approaches are developed and published. In the following, we attempt to summarize several major approaches and their relation to the modeling of postsynaptic proteins and their complexes.

### Protein structure prediction

**Fig. 2:**
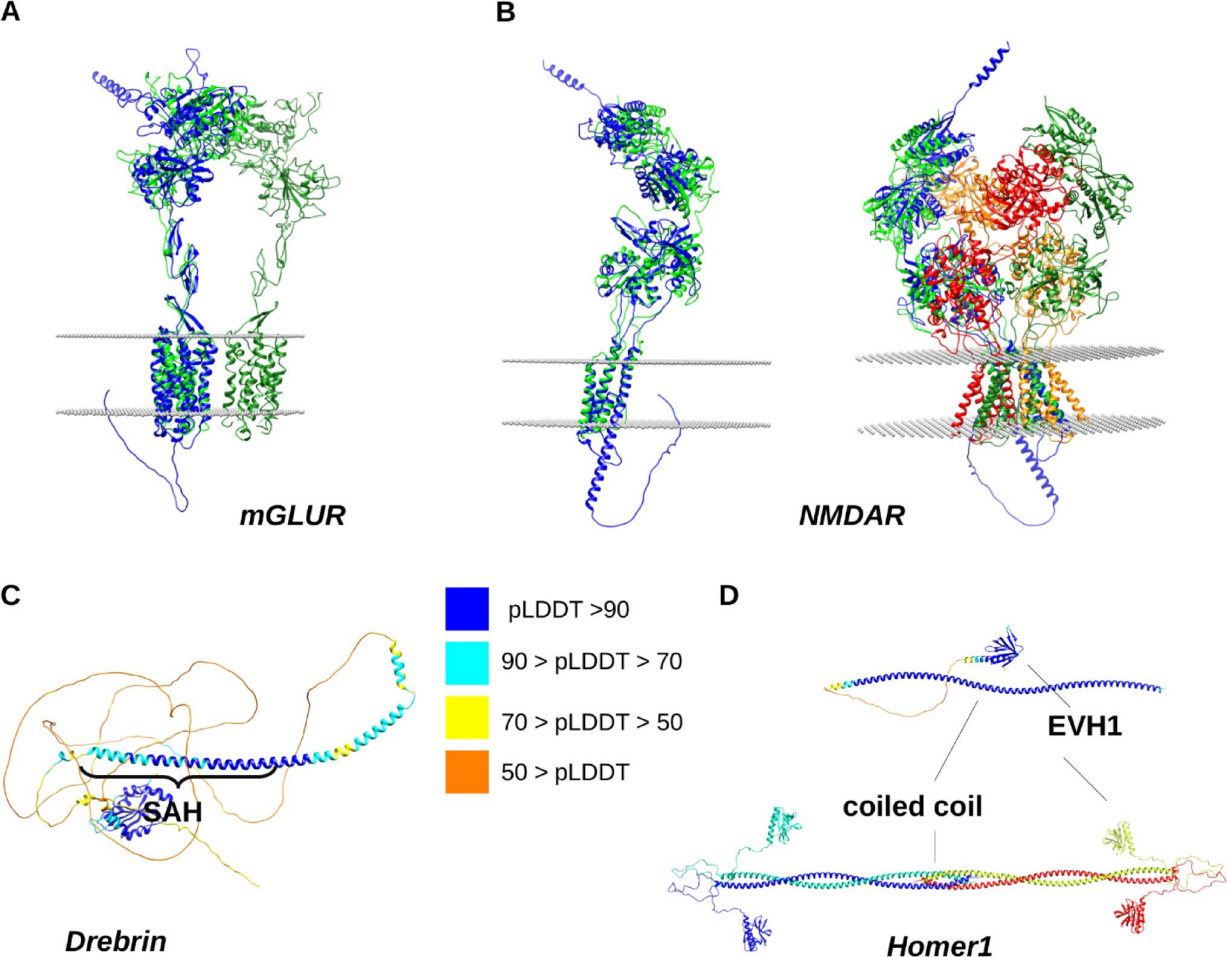
Examples of AlphaFold2 models of proteins in the postsynaptic density. **A** predicted (blue) and experimental (PDB 7WI6, green and dark green chains [36]) structure of human metabotropic glutamate receptor. The intracellular region protrudes into the membrane plane determined with TMDET [34]. **B** Predicted (blue) and experimental structures (PDB 5IOU; green, dark green orange and red chains [37]) of the *X. leavis* NMDA receptor. Both the monomer and the tetramer are shown for clarity. The membrane plane determined for the monomer does not fully match that determined for the experimental tetramer, but the transmembrane helices are largely correctly predicted. There are non-TM regions protruding into the membrane. **C** AF2-predicted structure of Drebrin, with the approximate position of the experimentally confirmed single alpha-helix (SAH) motif indicated. **D** AF2-predicted model of rat Homer1 as available in Uniprot (top). The architecture of Homer1 containing long coiled coil segments as deduced from the literature and the available experimental structure of the tetramerization region (PDB 3CVE [38]). The AF2 models in (**C** and **D**) are colored according to pLDDT score as shown in the color key. Structures were rendered and superimposed using Chimera [39]

The protein structure prediction problem is the one for which a major breakthrough was achieved with the development of AlphaFold2 (AF2) [25], recognized by the Nobel prize in Chemistry in 2024 [26]. Now, AlphaFold models are available in the PDB [27], in UniProt [28] and in the huge AlphaFold Protein Structure Database (AlphaFoldDB) with over 200 million entries [29]. This development also created a situation where the current best prediction methods are almost exclusively deep learning-based approaches. These currently range from multiple sequence alignment-based methods to language-based descriptions [30, 31] AlphaFold2 relies on an innovative pipeline optimizing local and global structural features in an iterative manner to obtain a consistent structural description, which is then translated to 3D coordinates. It should also be noted that AF2 relies on a template library and the templates available affect the prediction output – if experimentally determined structures for close homologs of the target protein are available, the prediction output is expected to resemble these templates. Nevertheless, AF2 opened a whole new era in structural biology, evidenced by the launch of the AlphaFold database and the inclusion of AF2 models in UniProt and even in the PDB. AF2 was primarily developed for monomeric globular proteins, and, as such, the claims that it has “solved the protein folding problem” in general were, while understandable, a bit exaggerated. There are specialized solutions to predict the structure of multidomain proteins, that outperform “simple” AF2-based predictions, like DeepAssembly, also applicable for multimeric transmembrane structures [32]. However, the variability of protein architectures makes it difficult to provide accurate predictions for cases where the molecules contain structural region types for which AF2 was not explicitly trained. For example, concerns arise for the predictions of transmembrane proteins, itself a diverse group. One of the most important differences within these groups is the number of transmembrane (TM) segments. Proteins with multiple TM helices such as G-protein coupled receptors (GPCRs) have a membrane-embedded structural part that is relatively compact and thus in many aspects resembles globular protein domains. For such proteins, AF2 predictions can be considered to work fairly well [33]. For single-pass membrane proteins, the TM region is a single alpha-helix, and the overall structure of the polypeptide chain is architecturally more similar to a modular protein with a non-globular segment. Importantly, AF2 was not trained to consider the presence and position of the lipid bilayer, thus, it can also not take the associated physical constraints into account. Therefore, in AF2 models some parts of the protein can protrude into the membrane even if this is physically and biologically unrealistic. The TmAlphaFold database is aimed at providing help for the evaluation of AF2-predicted structures of transmembrane proteins, by calculating the position of the membrane bilayer around the protein using TMDET [34], and identifying the protein parts that are unrealistically placed [35]. This kind of analysis is also useful for polypeptide chains that are actually part of multichain complexes in their biologically active form. For example, in the case of the AF2-predicted structure of human metabotropic glutamate receptor 3, the AF2 structure predicts the transmembrane core fairly well, probably because of the high number of templates available for GPCRs. Even though the receptor is dimeric, the monomeric structure recapitulates the TM region and the extracellular domains of the experimental structure (7WI6, [36]) well (Fig. 2A). For the tetrameric NMDA receptor, AF2 also correctly predicts the TM helices for a single monomer compared to the available structures (5IOU [37]). In both cases, only a small interference with a cytoplasmic disordered region with the membrane is observed (Fig. 2B).

Another issue to consider is the use of AF2 to predict the structure of full polypeptide chains. Many proteins, including the large scaffold proteins of the postsynaptic density, like PSD-95, GKAP, and members of the Shank, and Homer families, contain non-globular segments that require specific treatment. These non-globular segments can be intrinsically disordered or might form fibrillar structures, most often depending on the amino acid composition and repetitive nature of the underlying low-complexity sequence [40]. The recognition of intrinsically disordered segments as well as specific fibrillar motifs requires specialized tools, nowadays enhanced by deep learning-based approaches [41].

However, this also means that the validity of “pure” AF2 models can not easily be assessed without some preliminary knowledge on the actual protein. The proteins Drebrin and Homer1 can be brought as examples. For the actin binding postsynaptic protein Drebrin, AF2 predicts an almost fully disordered structure with the exception of the N-terminal ADF-H domain. In addition, a long alpha-helical region in the region 179–251 is also present in the AF2 structure with high confidence (pLDDT) scores. Actually, the segment 173–238, rich in charged amino acids arranged in a repetitive pattern, has been formerly predicted and recently shown experimentally to form a single alpha-helix (SAH) motif. SAHs are stable as monomers in solution [42], and specialized tools are required to discriminate them from coiled coil and disordered segments. For Drebrin, NMR chemical shifts also indicate that the helical region might indeed extend until residue 250 [43], in accordance with the helical structural preference predicted by AF2 (Fig. 2C). The prediction for Homer1 seems similar, with a globular N-terminal EVH1 domain, followed by a disordered region and a long alpha-helix, again predicted with high confidence. However, in this case the presence of a long monomeric helix is misleading, as this region is likely a coiled coil promoting the higher-order assembly of the protein, with its C-terminal region even characterized experimentally as a tetrameric coiled coil segment (PDB 3CVE [38]). Our own recent modeling study shows that the stability of the coiled coil structure substantially varies along the polypeptide chain, providing flexibility for the Homer1 scaffold even in its tetrameric, assembled state [44] (Fig. 2D). Thus, even if the local structural features, like high alpha-helical propensity, are correctly predicted for both Drebrin and Homer1, the exact nature of these non-globular segments can not be unambiguously deciphered from the monomeric structural models, which leads us to the next problem, multimer modeling.

### Predicting the structure of protein complexes

The dominance of AI-based methods in protein complex modeling is less pronounced than for the single protein structures. Of course, the number of deep learning-based approaches is still steadily growing in this area [45]. Following the success of AF2, it was immediately recognized that the same pipeline might be used to predict the structures of protein assemblies. First, AlphaFold-Multimer [46] was created, and lately, AlphaFold3 [47], focusing on protein complexes and protein:nucleic acid assemblies, was also published. These tools are direct competitors of more traditional methods like protein:protein docking approaches. Although there are approaches to predict the presence of the interactions between two or more partners [48], the methods we focus on here require the partner proteins as input, meaning that these tools can be used when the interaction itself is either experimentally described or predicted with confidence. One of the problems encountered here is the wide range of binding strengths between the partners. Stronger complexes (with lower dissociation constants) seem to be easier to predict than weak, transient ones [49]. The other, not entirely unrelated aspect is the nature of the binding region, whether it is globular, intrinsically disordered before partner binding, or actually forms a fibrillar structure. Currently no “magic tool” is available, that can handle all the different cases uniformly well, and, again, knowledge of the given protein and system is required. On the other hand, the realistic accessibility of the interacting regions should be modeled properly. For example, in the AF2 model of the NMDA receptor discussed above, the C-terminal cytoplasmic tail is buried in the membrane, whereas in reality, it should be available for interaction with PDZ domains of scaffold proteins. In another case, the coiled coil of Homer1 can not simply be modeled by putting the straight alpha-helices of the monomeric models next to each other as the expected structure is actually a left-handed supercoil. A not entirely unrelated aspect, at least from the modeling point of view, is the presence of structural transitions upon partner binding. Currently, induced fit can be considered during docking only by sophisticated multi-step pipelines [50].

Many proteins not only participate in binary interactions but are part of larger multimeric complexes. These can be generated from binary interactions in a stepwise manner, but of course, the potential inaccuracies in the binary assemblies are expected to propagate to the full assembly.

This also means that reliable data are needed both for the composition of synaptic complexes and binary interactions. The latter might not be sufficient to obtain from general databases, thus, a specialized database for postsynaptic interactions, PSINDB, was created by our group [51]. It not only contains the protein pairs but also the interacting regions, wherever available – this is especially important for the characterization of multivalent interactions. There are several AI-based tools to predict protein complexes based on protein:protein interaction network data [52, 53].

Computational prediction and analysis of possible complexes formed by a given set of proteins is yet another problem, which has particular significance for the “Lego-like” PSD protein pool. For this, simulations utilizing the Gillespie algorithm can be applied. One such tool is Cytocast, which has been already used to assess the different distribution of potential complexes in a simplified PSD model with different input protein abundances [54, 55]. These simulations highlighted the non-trivial relationship between protein availability, complex formation, and the presence of mutations weakening a given interaction.

#### Modeling structural flexibility

As the PSD contains a number of large scaffold proteins with substantial intrinsic disorder, accurate modeling of such segments is crucial for a structural description of this protein network. Currently, the most popular approach is ensemble-based modeling, for which a number of classical approaches are available along with recent AI-based tools like IDPForge [56]. The Protein Ensemble Database [57] contains multi-model structures of flexible protein segments that vary greatly in their number of conformers as well as the combination of experimental and computational methods with which they were obtained. The two most pressing problems are the generation of sufficient conformational variability, denoted proper conformational sampling, and ensuring the correspondence to the available experimental parameters, which are not necessarily easy to obtain. The conformational variability of intrinsically disordered segments is immense, and is still an actively explored problem using different kinds of conformer generator approaches [58], and advanced molecular dynamics techniques (e.g. [59]) using force fields specific for intrinsically disordered proteins (IDPs) [60]. A relatively simple approach is to generate a conformer pool and using this to select a minimal set of conformers that satisfy the available experimental data. The advantage of this is avoiding potential overfitting, as the number of conformers is not larger than absolutely necessary for the achieved correspondence with measurements. However, to model a more realistic scenario, obtaining a Boltzmann-weighted ensemble is desirable, where the conformations represented are weighted according to their relative occurrence during the actual structural fluctuations. Many current approaches thus target the generation of a Boltzmann ensemble, most often by reweighting. The goal of several ongoing deep learning-based developments is a method that can directly generate such ensembles. One of the major bottlenecks is the availability of suitable data for training and standardized evaluation [61]. Currently, a more promising approach is to use AI to assist with the sampling, that can range from generating starting structures to actually accelerating molecular dynamics based investigations by learning how to generate structures separated by larger time steps during a simulation, like the Timewarp approach [62]. A recently published method, Alphafold-Metainference, uses AF2-predicted distances to aid ensemble generation [63]. Yet another application of machine learning in molecular dynamics is the use of a forced field trained on accurate data obtained with quantum chemical calculations. AI2BMD is an implementation of this principle that uses protein fragments to tackle the high computational demand of the calculations needed to generate the training data [64].

**Fig. 3:**
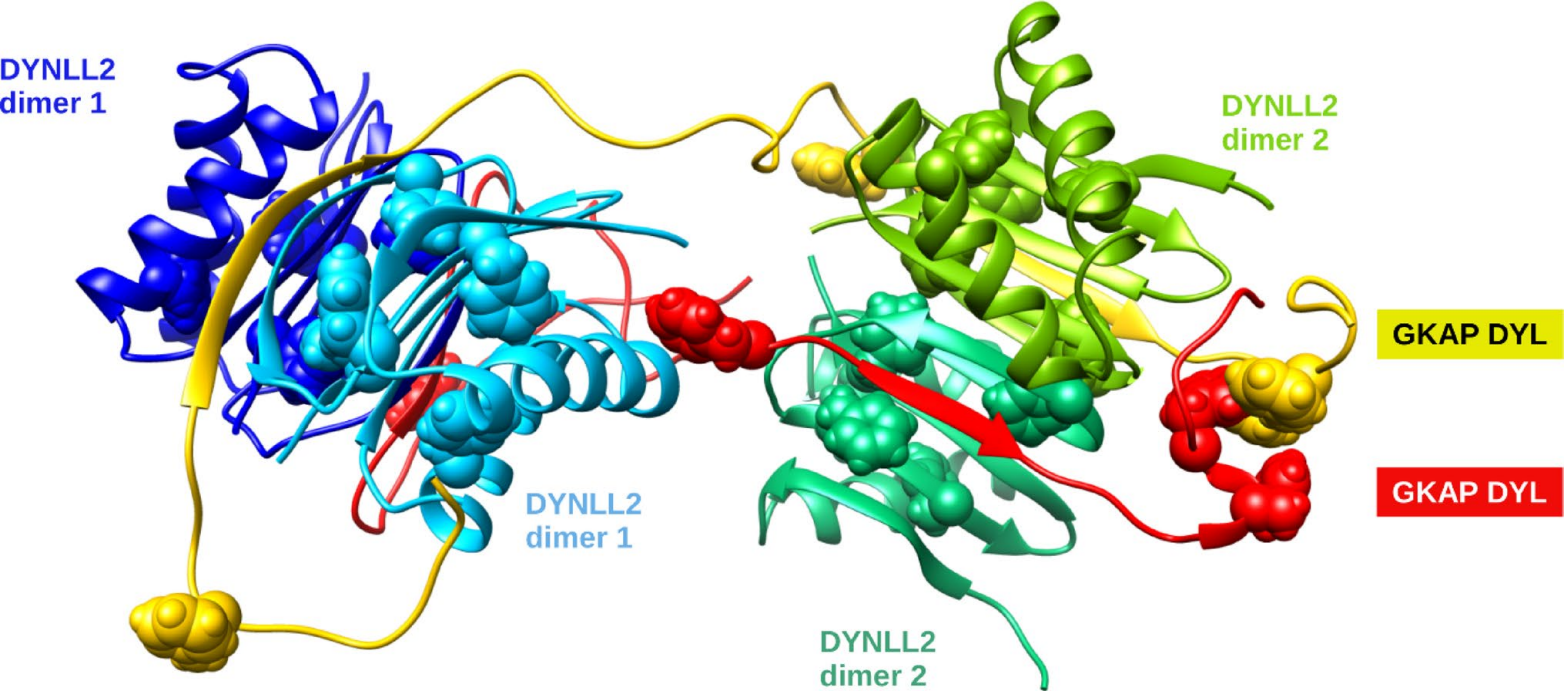
Snapshot from a molecular dynamics simulation of the multivalent GKAP:LC8 complex. The GKAP chains, depicted in red and yellow, retain their flexibility between their LC8-binding sites, allowing LC8 dimers (in cyan/blue and green/dark green) to adopt various mutual orientations even in their bound state. Spacefill representations indicate aromatic residues potentially involved in intermolecular contacts outside the classical GKAP:LC8 binding site. Figure prepared with Chimera [39]

Another challenge is the prediction of the degree of flexibility in protein complexes. Many PSD proteins contain disordered binding regions, oftentimes in a tandem configuration, allowing multivalent binding. Currently, such systems can primarily be explored by traditional molecular dynamics simulations, and the role of AI is confined to the suggestion of initial conformations. A recent example of such a system is the multivalent complex between the almost entirely disordered GKAP scaffold protein and two dimers of the globular LC8 hub protein. NMR spectroscopic data indicate that the linkers between the LC8-binding motifs remain largely disordered in the complex, and molecular dynamics simulations are consistent with this observation. A number of possible complex conformations were also generated using ColabFold [65], and these also suggest that the complex can adopt various overall structures with different relative orientations of the LC8 dimers (Fig. 3). These conformations were also used as starting conformations for molecular dynamics calculations [66].

#### Prediction of phase separation

Protein phase separation is a relatively recently recognized biomolecular phenomenon. Proteins undergoing phase separation form dense condensates, also often termed membraneless organelles (MLOs) because of their complexity. Such condensates provide high local concentrations for the proteins while also providing a well-separated environment for biochemical processes. Because of their membraneless nature, MLOs can be formed and dissolved rapidly. The molecular features allowing phase separation are still not completely understood, complicated by the existence of driver proteins that can initiate condensation, and passengers, that can be included in the already formed MLOs but are not capable of promoting the process on their own. The types of interactions important in phase separation are in principle the same as recognized for protein folding, the difference lies in their patterning [67]. A key aspect, however, seems to be multivalency, probably allowing the proximity of molecules while the actual interactions are dynamically changing between them – a phenomenon previously described for “fuzzy complexes”, as exemplified by the FuzDB database [68].

A number of postsynaptic proteins, including major scaffolds, have been shown to form condensates [23, 69, 70] in various configurations. Recently, the role of a microexon-encoded peptide segment in the mRNA-regulatory CPEB4 protein was shown to mediate the dissolution of condensates upon neuron activation via the slight pH change accompanying the stimulus. Decreased inclusion of this exon, as occurs in ASD, leads to more stable condensates. The key interaction identified here is between an arginine-rich stretch in the microexon-encoded peptide and a histidine cluster located before it [24]. This mechanism is an example of translation-level regulation modulated by synaptic activity.

Such observations highlight the importance of predicting LLPS formation, even in a condition-dependent manner. Here, not only the propensity of a given protein to be included in condensates is key, but also the exact residues promoting this behavior. The recently developed machine learning-based PSPHunter method can predict the residues responsible for phase separation [71].

#### Analysis of the effect of mutations

Last but not least, a structure-based model of the postsynapse should also be able to account for the effect of posttranslational protein modifications (PTMs) and mutations. Posttranslational modifications are common in most eukaryotic proteins, and the postsynapse is no exception. One task is to predict the sites and nature of PTMs, another is to decipher their effect on protein:protein interactions. In the PSD, activity-dependent PTMs are highly likely to modulate the postsynaptic nanostructure [16].

For the prediction of PTMs, a number of machine learning-based approaches are already available, for example for phosphorylation, acetylation and ubiquitination, many of these published after 2019 [72]. These are all specialized tools, meaning that no general method capable of predicting multiple types of PTMs of a given protein seems to be available.

Understanding the molecular basis of cognitive functions and their alteration should also include accurate prediction of the effect of mutations on protein:protein association rates, a task that has been addressed intensively in the past decade but no accurate general solution has been achieved yet.

The success of AlphaFold2 raised the hypothesis that maybe predicted structures can reveal the effect of mutations on structures. However, these expectations should be treated with caution [73]. It can be noted here that functionally relevant structural variability might be predicted by using selected subsets of the multiple sequence alignment used as an input [74]. However, a non-negligible aspect is that because of the template-based nature of AF2, a mutant protein will likely have a predicted structure closely matching the template with highest sequence similarity, if any. Still, for proteins with no available experimental structure, an AF2 model might be valuable in providing a structural context for the mutated positions. This concept is exploited in the workflow of the variant effect predictor AlphaMissense, combined with protein language modeling [75].

Another approach is the competitive binding assay based on AlphaFold. Here, two binding partners, typically peptides, are presented for a given protein, and the AF2-generated complex is expected to contain the stronger binder. This method is expected to be able to rank peptides based on binding affinity to a given partner, and this, even discriminate between mutant variants [76].

## Current solutions for the computational modeling of postsynaptic complexes and future directions

As enumerated above, there are many solutions in structural biology that can tackle parts of the modeling aspects of highly complex macromolecular assemblies. These increasingly rely on deep learning-based approaches. However, the nature of the postsynaptic protein network well illustrates that there is still a long way to go to get a realistic, atomic-level description of a highly elaborate molecular machinery. Here we highlight the strategies available with current tools and envision some foreseeable developments in the field. This overview, however, is rather illustrative than exhaustive, and is centered around the understanding of the molecular assemblies and events in the postsynapse.

### Realistic structural models of modular proteins

The current approach to model a protein with distinct structural parts is a multistep process: first, the distinct parts should be identified, then, each of these should be modeled with appropriate – if needed, specialized – tools, then reassembled in a way that provides a realistic overall structure. This is the approach we followed in reconstructing the Homer1 tetramer [44], where the coiled coil segment was modeled with CCBuilder [77] and Isambard [78], the disordered part with DIPEND [79], and these segments were then combined with the available experimental structures of the globular EVH1 domain. This approach has previously been used for other protein assemblies with long coiled coil segments, like myosin 2 [80]. Such an approach needs much manual intervention along with a preliminary concept based on literature data and conventional sequence-based predictions. The sites where the fragments are combined also need special attention. A logical future direction could be an implementation of an automated or at least semi-automated approach that iteratively performs the identification of segments with different properties, their first-approximation modeling, and then refines the initial models using considerations on the global structure. Although it can not be expected that multimerization state can properly be recognized during such a pipeline, it could be capable of providing feedback on the regions that might participate in obligate homo- or heteromeric interactions, coiled coils being a prime example of these.

Transmembrane protein modeling can also fit into such a pipeline – once the transmembrane nature of a protein and the TM regions are identified, constraints could be applied to ensure that no other parts interfere with the lipid bilayer.

Naturally, all these aspects could in principle be implemented using deep learning, similar to the concept of iterative optimization and reconciliation of local and global structural features in AlphaFold2. The generation of physically realistic structures is an aspect that is already among the major goals of the latest generative AI tools like AlphaFold3, which are prone to “hallucinate” [47]. Thus, the integration of (bio)physical knowledge is inevitable for the next generation of deep learning-based structure prediction tools to obtain realistic models of full-length proteins.

### Modeling the flexibility of macromolecular assemblies

Modeling macromolecular assemblies should explicitly aim at exploring the possible assembly modes and conformations for a given complex. While this aspect is already present and made use of in AlphaFold-assisted ensemble generation and complex modeling, it should be an integral part of the process. The current approaches combine initial structure generation and molecular dynamics simulations, or, generating possible conformers and performing molecular docking on the different structures. Combining initial data generation with AI approaches capable of mimicking long molecular dynamics (MD) simulations like Timewarp [62], and performing an iterative modeling procedure, could largely enhance the sampling of possible conformations of protein complexes. Of course, similar to the generation of ensembles of individual segments, methods that directly output an ensemble-based description of a multicomponent complex could also be envisioned. Although such methods could in principle be trained using conformer sets obtained from molecular dynamics, the amount of data is currently scarce for monomeric systems, and it might not be feasible in the near future to scale up the efforts required to overcome this bottleneck. Moreover, in this field, the availability of experimental measurements that can be used for benchmarking is also a practical limitation. Larger complexes can routinely be investigated by X-ray or cryo-EM at the atomic level, providing little direct information on their dynamics. NMR spectroscopy is only suitable to investigate the parts that remain largely flexible and in many cases, details of the interactions between slowly tumbling structured domains remain indiscernible. Small-angle X-ray scattering can be used in solution but it does not provide direct information for individual atoms or residues. Nevertheless, it is rightly expected that the modeling of multiple conformational states of larger protein complexes will benefit from AI-based approaches.

### Functional relevance of macromolecular complexes at the scale of the full PSD–also in the light of mutations

A striking feature of many mutations causing cognitive disorders is their relatively confined effect as most neural functions remain intact despite the mutations affecting abundant postsynaptic proteins. This might indicate that these changes cause relatively subtle perturbations in the postsynaptic protein organization, and it also might be that these either occur or cause functional alterations in only a small population of cells. Our recent Cytocast-based simulation indicates strong context-dependence in the effect of an interaction-weakening mutation [55]. However, our model is a highly simplified one, and to obtain clinically relevant hypotheses, more complex, and thus, more realistic models are required, that take into account all relevant proteins, membrane-associated localization, as well as realistic 3D conformations of the partners and complexes. This last aspect is quite important as the 3D organization determines the availability of binding sites for novel interactions.

Modeling phase-separated condensates is currently only possible with molecular dynamics simulations of simplified yet huge systems for which specialized supercomputers could be used [81]. The problem is that the modeled system should contain a large number of copies of the constituent protein(s) to obtain results relevant for the entire separated phase. Nevertheless, the mere fact that such studies can in principle be done provides the hope for the emergence of approaches that can tackle the phenomenon of phase separation, and here the role of AI-accelerated simulations could also be paramount.

Many computational neuron models focus on the electrochemical behavior of these cells, from the generation to the propagation of action potentials. There are only a few efforts to integrate such models with the molecular events occurring within the PSD network, mostly because their functional connection is largely elusive. However, a more complete description of neural function should inevitably include the postsynaptic machinery and take into account its short- and long-term reorganization based on stimuli [82]. Many neural models are already compartmentalized and contain parameters [83] related to the density of certain transmembrane receptors, mostly ion channels. Better understanding the organization and function of synaptic nanodomains could lead to the refinement of these models.

### Integration with experimental data

So far, we only touched the relationship between models and experimental data obtained by *in vitro* structural investigations. While the use of different measured parameters is an integral part of conventional structure determination, complex modeling and ensemble generation approaches, most contemporary AI-based approaches use experimental data for refinement and/or validation of computer-generated models. Structural models of individual protein components can also be fit with experimental data obtained for large assemblies like demonstrated on the nuclear pore complex [84]. We argue that the direct use of experimental information by deep learning-based approaches as an input is a logical next step and could enhance the self-consistency of structural models at different scales, from individual protein structures to large complexes. In the field of NMR spectroscopy, AI-assisted pipelines already exist that incorporate the processing and interpretation of the measured spectra and provide an automated way to calculate structures from these [85]. For example, inter-residue distances from fluorescent resonance energy transfer or chemical cross-linking experiments could in principle be used to constrain the generated assemblies. Here, the practical problem to be solved is the encoding of such data in a way that could be fed into deep learning-based methods, but there is already precedent to use distance maps or orientograms as inputs for different parts of complex approaches. Again, AlphaFold2 can be mentioned as an example as the structural description generated by its EvoFormer module contains a ‘pair representation’ conceptually similar to such data.

We also would like to argue here that low-resolution ultrastructural data obtained from various microscopic techniques, as well as the neural response to site-specific excitation of neurons by uncaging and infrared stimulation should be integral part of the detailed synaptic models on the long term. In this respect, the combination of models with in vivo data like protein localization [86] and multi-modal measurements [87], is a major next step towards the reconstruction of physiologically relevant macromolecular assemblies at the scale of the full PSD.

The ultimate goal, of course, would be to move from the models of individual postsynaptic regions to the description of full neurons that might contain thousands of dendritic spines. Eventually, the description of larger neural networks could also benefit from the lessons learned from the study of nanostructures in the PSD, and the ultimate test of such models will come from the comparison with experimental data.

## Conclusions and outlook

Deep learning-based approaches (examples highlighted in Table 1) are already an important part of structural biology research, integrated into and/or complementing exsiting major databases (Table 2). The current state of the art is that there are various tools available for a number of individual problems, but the performance and availability of these depends heavily on the specific question. Here we provided the example of the postsynaptic density as an overly complex, yet organized protein network that is especially hard to investigate at the molecular level in its entirety. As the major strength of AI is to crack the challenges raised by such level of complexity, it might not be overly optimistic to expect that future developments will allow the description of the postsynaptic protein network in sufficient detail.

**Table 1:** Summary of selected AI-based tools for protein structure prediction/generation discussed in the text

Name	Short description	Performance/usage notes	References
AlphaFold2 (AF2)	State-of-the-art protein structure prection method	Was developed for, thus performs best on monomeric globular structures	[25]
AlpaFold-Multimer	AF2-based tool for modeling protein complexes	Needs explicit input on the stoichiometry of the complex	[38]
ColabFold	Community-accessible interface to generate AF2 models	Needs proper parametrization	[65]
AlphaFold3	Next-generation general structure prediction method with focus on assemblies	Generative AI, prone to “hallucinate” physically unrealistic details	[47]
DeepAssembly	Predicts multidomain and multimeric structures, including transmembrane proteins	Available structures for training might not capture sufficient diversity e.g. for transmembrane proteins	[32]
IDPForge	Tool to generate conformational ensembles of disordered segments	Might need more and experimentally verified ensembles for training to capture difficult cases	[56]
Timewarp	Accelerates molecular dynamics calculations by estimating characteristic motions	Might need more and experimentally verified ensembles for training to describe diverse systems	[62]
PSPHunter	Prediction of regions/residues key in phase separation	Might need more experimental data for training for more accurate prediction of phase separating proteins	[71]
AlphaMissense	Prediction of the effect of mutations using AF2-derived structural context	Inaccurate AF2 predictions might influence expected structural context	[75]

**Table 2:** Selected databases relevant in structure prediction

Database	Description	References
PDB	The primary protein structure database, contains experimentally determined models plus computed ones, including AF2-derived ones	[27]
UniProt	Comprehensive protein sequence database, includes AF2-based models for full polypeptide chains	[28]
AlphaFoldDB	Large database of AF2-predicted structural models, including predictions for complete proteomes	[29]
TMAlphaFold	Structures and evaluation of AF2-derived models for transmembrane proteins	[35]
Protein Ensemble Database	Contains structural ensembles for flexible protein and segments determined using various combinations of experimental and computational methods	[57]

Protein structures posed such a seemingly impossible challenge, first for experimentalists, then for computers, although technical developments suggested that the problem is not unsolvable per se. John Kendrew’s work coincided with the emergence of computers necessary for data processing, and this provided a turning point in protein structure determination despite the skepticism of his contemporaries [88]. The success of AlphaFold2 was also not “out of the blue”, although it occurred sooner than many structural biologists anticipated based on the pace at which the accuracy of predictions grew. We envision a similar scenario for the investigation of complex molecular machineries: the amount of available data grows along with the diversity of AI-based approaches, and we might well arrive at the point when a suitable combination of the aspects discussed in this review can be used in an innovative integrated approach. Given the pace of AI development, this turning point could be closer than we can anticipate, and it could help us decipher the mechanisms behind an even more complex natural intelligence, our own brain.

## References

[1] Poldrack RA, Farag MJ. Progress and challenges in probing the human brain. Nature. 2015. 10.1038/nature15692. 10.1038/nature15692 26469048

[2] Krauss P. The most complex system in the universe. In: Artificial intelligence and brain research. Springer, Berlin, Heidelberg; 2023. pp 15–18. 10.1007/978-3-662-68980-6_2.

[3] Stevens CF. The neuron. Sci Am. 1979. 10.1038/scientificamerican0979-54.

[4] Hobert O, Carrera I, Stefanakis N. The molecular and gene regulatory signature of a neuron. Trends Neurosci. 2010. 10.1016/j.tins.2010.05.006. 10.1016/j.tins.2010.05.006 PMC2947585 20663572

[5] Abriata LA. The Nobel Prize in chemistry past, present, and future of AI in biology. Commun Biol. 2024. 10.1038/s42003-024-07113-5. 10.1038/s42003-024-07113-5 PMC11522274 39472680

[6] Rosignoli S, Pacelli Mi, Manganiello F, Paiardini A. An outlook on structural biology after AlphaFold tools, limits and perspectives. FEBS Open Bio. 2025. 10.1002/2211-5463.13902. 10.1002/2211-5463.13902 PMC11788754 39313455

[7] Seguin C, Sporns O, Zalesky A. Brain network communication: concepts, models and applications. Nat Rev Neurosci. 2023. 10.1038/s41583-023-00718-5. 10.1038/s41583-023-00718-5 37438433

[8] Piwecka M, Rajewsky N, Rybak-Wolf A. Single-cell and spatial transcriptomics: deciphering brain complexity in health and disease. Nat Rev Neurol. 2023. 10.1038/s41582-023-00809-y. 10.1038/s41582-023-00809-y PMC10191412 37198436

[9] Grant SGN. Synapse molecular complexity and the plasticity behaviour problem. Brain Neurosci Adv. 2018. 10.1177/2398212818810685. 10.1177/2398212818810685 PMC7058196 32166154

[10] Verpelli C, Sala C. Molecular and synaptic defects in intellectual disability syndromes. Curr Opin Neurobiol. 2012. 10.1016/j.conb.2011.09.007. 10.1016/j.conb.2011.09.007 22000839

[11] Verpelli C, Montani C, Vicidomini C, Heise C, Sala C. Mutations of the synapse genes and intellectual disability syndromes. Eur J Pharmacol. 2013. 10.1016/j.ejphar.2013.07.023. 10.1016/j.ejphar.2013.07.023 23872408

[12] Grant SGN. The synaptomic theory of behavior and brain disease. Cold Spring Harb Symp Quant Biol. 2019. 10.1101/sqb.2018.83.037887. 10.1101/sqb.2018.83.037887 30886054

[13] Bailey CH, Kandel ER. Synaptic remodeling, synaptic growth and the storage of long-term memory in Aplysia. Prog Brain Res. 2008. 10.1016/S0079-6123(07)00010-6. 10.1016/S0079-6123(07)00010-6 18394474

[14] Bourne JN, Harris KM. Coordination of size and number of excitatory and inhibitory synapses results in a balanced structural plasticity along mature hippocampal CA1 dendrites during LTP. Hippocampus. 2011. 10.1002/hipo.20768. 10.1002/hipo.20768 PMC2891364 20101601

[15] Alvarez-Castelao B, Schuman EM. The regulation of synaptic protein turnover. J Biol Chem. 2015. 10.1074/jbc.R115.657130. 10.1074/jbc.R115.657130 PMC4661377 26453306

[16] Droogers WJ, MacGillavry HD. Plasticity of postsynaptic nanostructure. Mol Cell Neurosci. 2023. 10.1016/j.mcn.2023.103819. 10.1016/j.mcn.2023.103819 36720293

[17] Tang AH, et al. A trans-synaptic nanocolumn aligns neurotransmitter release to receptors. Nature. 2016. 10.1038/nature19058. 10.1038/nature19058 PMC5002394 27462810

[18] Manning A, et al. Trans-synaptic association of vesicular zinc transporter 3 and Shank3 supports synapse-specific dendritic spine structure and function in the mouse auditory cortex. J Neurosci. 2024. 10.1523/jneurosci.0619-24.2024. 10.1523/JNEUROSCI.0619-24.2024 PMC11236586 38830758

[19] Parisi G, Palopoli N, Tosatto SCE, Fornasari MS, Tompa P. “Protein” no longer means what it used to. Curr Res Struct Biol. 2021. 10.1016/j.crstbi.2021.06.002. 10.1016/j.crstbi.2021.06.002 PMC8283027 34308370

[20] Verpelli C, Schmeisser MJ, Sala C, Boeckers TM. Scaffold proteins at the postsynaptic density. In: Kreutz M, Sala C, editors. Synaptic plasticity. Advances in experimental medicine and biology, vol. 970. Springer, Vienna; 2012. pp. 29–61. 10.1007/978-3-7091-0932-8_2. 10.1007/978-3-7091-0932-8_2 22351050

[21] Roy M, Sorokina O, Skene N, Simonnet C, Mazzo F, Zwart R, et al. Proteomic analysis of postsynaptic proteins in regions of the human neocortex. Nat Neurosci. 2018. 10.1038/s41593-017-0025-9. 10.1038/s41593-017-0025-9 29203896

[22] Kiss-Tóth A, Dobson L, Péterfia B, Ángyán AF, Ligeti B, Lukács G, et al. Occurrence of ordered and disordered structural elements in postsynaptic proteins supports optimization for interaction diversity. Entropy. 2019. 10.3390/e21080761. 10.3390/e21080761 PMC7515291 33267475

[23] Chen X, Wu X, Wu H, Zhang M. Phase separation at the synapse. Nat Neurosci. 2020. 10.1038/s41593-019-0579-9. 10.1038/s41593-019-0579-9 32015539

[24] Garcia-Cabau C, Bartomeu A, Tesei G, Cheung KC, Pose-Utrilla J, Picó S, et al. Mis-splicing of a neuronal microexon promotes CPEB4 aggregation in ASD. Nature. 2025. 10.1038/s41586-024-08289-w. 10.1038/s41586-024-08289-w PMC11711090 39633052

[25] Jumper J, et al. Highly accurate protein structure prediction with AlphaFold. Nature. 2021. 10.1038/s41586-021-03819-2. 10.1038/s41586-021-03819-2 PMC8371605 34265844

[26] Paiva S. Protein prediction takes the prize. Nat Chem. 2024. 10.1038/s41557-024-01699-3. 10.1038/s41557-024-01699-3 39622941

[27] Berman HM, Westbrook J, Feng Z, Gilliland G, Bhat TN, Weissig H, et al. The Protein Data Bank. Nucleic Acids Res. 2000. 10.1093/nar/28.1.235. 10.1093/nar/28.1.235 PMC102472 10592235

[28] The UniProt Consortium. Uniprot: the universal protein knowledgebase in 2023. Nucleic Acids Res. 2023. 10.1093/nar/gkac1052. 10.1093/nar/gkac1052 PMC9825514 36408920

[29] Varadi M, et al. AlphaFold protein structure database in 2024: providing structure coverage for over 214 million protein sequences. Nucleic Acids Res. 2024. 10.1093/nar/gkad1011. 10.1093/nar/gkad1011 PMC10767828 37933859

[30] Jänes J, Beltrao P. Deep learning for protein structure prediction and design—progress and applications. Mol Syst Biol. 2024. 10.1038/s44320-024-00016-x. 10.1038/s44320-024-00016-x PMC10912668 38291232

[31] Park S, Myung S, Bae M. Advancing protein structure prediction beyond AlphaFold2. Curr Opin Struct Biol. 2025. 10.1016/j.sbi.2025.102985. 10.1016/j.sbi.2025.102985 39862760

[32] Xia Y, Zhao K, Liu D, Zhou X, Zhang G. Multi-domain and complex protein structure prediction using inter-domain interactions from deep learning. Commun Biol. 2023. 10.1038/s42003-023-05610-7. 10.1038/s42003-023-05610-7 PMC10692239 38040847

[33] Hegedűs T, Geisler M, Lukács GL, Farkas B. Ins and outs of AlphaFold2 transmembrane protein structure predictions. Cell Mol Life Sci. 2022. 10.1007/s00018-021-04112-1. 10.1007/s00018-021-04112-1 PMC8761152 35034173

[34] Tusnády GE, Dosztányi Zs, Simon I. TMDET: web server for detecting transmembrane regions of proteins by using their 3D coordinates. Bioinformatics. 2005. 10.1093/bioinformatics/bti121. 10.1093/bioinformatics/bti121 15539454

[35] Dobson L, Szekeres LI, Gerdán C, Langó T, Zeke A, Tusnády GE. Tmalphafold database: membrane localization and evaluation of AlphaFold2 predicted alpha-helical transmembrane protein structures. Nucleic Acids Res. 2023. 10.1093/nar/gkac928. 10.1093/nar/gkac928 PMC9825488 36318239

[36] Fang W, et al. Structural basis of the activation of metabotropic glutamate receptor 3. Cell Res. 2022. 10.1038/s41422-022-00623-z. 10.1038/s41422-022-00623-z PMC9253128 35236939

[37] Zhu S, Stein RA, Yoshioka C, Lee CH, Goehring A, Mchaourab HS, et al. Mechanism of NMDA receptor inhibition and activation. Cell. 2016. 10.1016/j.cell.2016.03.028. 10.1016/j.cell.2016.03.028 PMC4914038 27062927

[38] Hayashi MK, Tang C, Verpelli C, Narayanan R, Stearns MH, Xu RM, et al. The postsynaptic density proteins Homer and Shank form a polymeric network structure. Cell. 2009. 10.1016/j.cell.2009.01.050. 10.1016/j.cell.2009.01.050 PMC2680917 19345194

[39] Pettersen EF, Goddard TD, Huang CC, Couch GS, Greenblatt DM, Meng EC, et al. UCSF chimera—a visualization system for exploratory research and analysis. J Comput Chem. 2004. 10.1002/jcc.20084. 10.1002/jcc.20084 15264254

[40] Mier P, Paladin L, Tamana S, Petrosian S, Hajdu-Soltész B, Urbanek A, et al. Disentangling the complexity of low complexity proteins. Brief Bioinform. 2020. 10.1093/bib/bbz007. 10.1093/bib/bbz007 PMC7299295 30698641

[41] Erdos G, Dosztanyi Z. Deep learning for intrinsically disordered proteins: From improved predictions to deciphering conformational ensembles. Curr Opin Struct Biol. 2025. 10.1016/j.sbi.2024.102950. 10.1016/j.sbi.2024.102950 39522439

[42] Varga S, Péterfia BF, Dudola D, Farkas V, Jeffries CM, Permi P, et al. Dynamic interchange of local residue–residue interactions in the largely extended single alpha-helix in Drebrin. Biochem J. 2025. 10.1042/BCJ20253036. 10.1042/BCJ20253036 PMC12203971 40192062

[43] Varga S, Kaasen JM, Gáspári Z, Péterfia B, Mulder F. Resonance assignment of the intrinsically disordered actin-binding region of Drebrin. Biomol NMR Assign. 2025. 10.1007/s12104-025-10239-0. 10.1007/s12104-025-10239-0 PMC12513958 40515930

[44] Kalman ZE, Czajlik A, Maruzs B, Farkas F, Pap I, Homonnay C, et al. Structural modelling and dynamics of the full-length Homer1 multimer. bioRxiv. 2025. 10.1101/2025.05.26.655084. 10.1002/prot.70091 PMC12954376 41267651

[45] Csikász-Nagy A, Fichó E, Noto S, Reguly I. Computational tools to predict context-specific protein complexes. Curr Opin Struct Biol. 2024. 10.1016/j.sbi.2024.102883. 10.1016/j.sbi.2024.102883 38986166

[46] Evans R, et al. Protein complex prediction with AlphaFold-Multimer. bioRxiv. 2022. 10.1101/2021.10.04.463034.

[47] Abramson J, et al. Accurate structure prediction of biomolecular interactions with AlphaFold 3. Nature. 2024. 10.1038/s41586-024-07487-w. 10.1038/s41586-024-07487-w PMC11168924 38718835

[48] Zhang J, Durham J, Cong Q. Revolutionizing protein–protein interaction prediction with deep learning. Curr Opin Struct Biol. 2024. 10.1016/j.sbi.2024.102775. 10.1016/j.sbi.2024.102775 38330793

[49] Burke DF, Bryant P, Barrio-Hernandez I, Memon D, Pozzati G, Shenoy A, et al. Towards a structurally resolved human protein interaction network. Nat Struct Mol Biol. 2023. 10.1038/s41594-022-00910-8. 10.1038/s41594-022-00910-8 PMC9935395 36690744

[50] Miller EB, Murphy RB, Sindhikara D, Borrell KW, Grisewood MJ, Fi R, et al. Reliable and accurate solution to the induced fit docking problem for protein−ligand binding. J Chem Theory Comput. 2021. 10.1021/acs.jctc.1c00136. 10.1021/acs.jctc.1c00136 33779166

[51] Kalman ZE, Dudola D, Mészáros B, Gáspári Z, Dobson L. PSINDB: the postsynaptic protein–protein interaction database. Database. 2022. 10.1093/database/baac007. 10.1093/database/baac007 PMC9216581 35234850

[52] Palukuri MV, Marcote EM. Super complex: a supervised machine learning pipeline for molecular complex detection in protein-interaction networks. PLoS ONE. 2021. 10.1371/journal.pone.0262056. 10.1371/journal.pone.0262056 PMC8719692 34972161

[53] Pan Y, Wang Y, Guan J, Zhou S. PCGAN: a generative approach for protein complex identification from protein interaction networks. Bioinformatics. 2023. 10.1093/bioinformatics/btad473. 10.1093/bioinformatics/btad473 PMC10457665 37531266

[54] Miski M, Keömley-Horváth BM, Rákóczi Megyeriné D, Csikász-Nagy A, Gáspári Z. Diversity of synaptic protein complexes as a function of the abundance of their constituent proteins: a modeling approach. PLoS Comput Biol. 2022. 10.1371/journal.pcbi.1009758. 10.1371/journal.pcbi.1009758 PMC8797218 35041658

[55] Miski M, Weber Á, Fekete-Molnár K, Keömley-Horváth BM, Csikász-Nagy A, Gáspári Z. Simulated complexes formed from a set of postsynaptic proteins suggest a localised effect of a hypomorphic Shank mutation. BMC Neurosci. 2024. 10.1186/s12868-024-00880-1. 10.1186/s12868-024-00880-1 PMC11227168 38971749

[56] Zhang O, Zi Liu ZH, Forman-Kay JD, Head-Gordon T. Deep learning of proteins with local and global regions of disorder. arXiv 2025; 10.48550/arXiv.2502.11326.

[57] Ghafouri H, et al. PED in 2024: improving the community deposition of structural ensembles for intrinsically disordered proteins. Nucleic Acids Res. 2024. 10.1093/nar/gkad947. 10.1093/nar/gkad947 PMC10767937 37904608

[58] Teixeira JMC, Liu ZH, Namini A, Li J, Vernon RM, Krzeminski M, et al. Idpconformergenerator: a flexible software suite for sampling the conformational space of disordered protein states. J Phys Chem A. 2022. 10.1021/acs.jpca.2c03726. 10.1021/acs.jpca.2c03726 PMC9465686 36030416

[59] Koneru JK, Reid KM, Robustelli, P. Performing all-atom molecular dynamics simulations of intrinsically disordered proteins with replica exchange solute tempering. arXiv 2025; 10.48550/arXiv.2505.01860.

[60] Muhammedkutty FNK, MacAinsh M, Zhou H-X. Atomistic molecular dynamics simulations of intrinsically disordered proteins. Curr Opin Struct Biol. 2025. 10.1016/j.sbi.2025.103029. 10.1016/j.sbi.2025.103029 PMC12360891 40068541

[61] Aranganathan A, Gu X, Wang D, Vani BP, Tiwary P. Modeling Boltzmann-weighted structural ensembles of proteins using artificial intelligence–based methods. Curr Opin Struct Biol. 2025. 10.1016/j.sbi.2025.103000. 10.1016/j.sbi.2025.103000 PMC12011212 39923288

[62] Klein L, Foong A, Fjelde T, Mlodozeniec B, Brockschmidt M, Nowozin S, et al. Timewarp: transferable acceleration of molecular dynamics by learning time-coarsened dynamics. Adv Neural Inf Process Syst. 2024;36:52863–83.

[63] Brotzakis ZF, Zhang S, Murtada MH, Vendruscolo M. AlphaFold prediction of structural ensembles of disordered proteins. Nat Commun. 2023. 10.1038/s41467-025-56572-9. 10.1038/s41467-025-56572-9 PMC11829000 39952928

[64] Wang T, He X, Li M, Li Y, Bi R, Wang Y, et al. Ab initio characterization of protein molecular dynamics with AI2BMD. Nature. 2024. 10.1038/s41586-024-08127-z. 10.1038/s41586-024-08127-z PMC11602711 39506110

[65] Mirdita M, Schütze K, Moriwaki Y, Heo L, Ovchinnikov S, Steinegger M. Colabfold: making protein folding accessible to all. Nat Methods. 2022. 10.1038/s41592-022-01488-1. 10.1038/s41592-022-01488-1 PMC9184281 35637307

[66] Nagy-Kanta E, Kálmán ZE, Tossavainen H, Juhász T, Farkas F, Hegedüs J, et al. Residual flexibility in the topologically constrained multivalent complex between the GKAP scaffold and LC8 hub proteins. FEBS J. 2025. 10.1111/febs.70219. 10.1111/febs.70219 PMC12797016 40843979

[67] Lin Y-H, Brady JP, Forman-Kay JD, Chan HS. Charge pattern matching as a ‘fuzzy’ mode of molecular recognition for the functional phase separations of intrinsically disordered proteins. New J Phys. 2017. 10.1088/1367-2630/aa9369.

[68] Hatos A, Monzon AM, Tosatto SCE, Piovesan D, Fuxreiter M. Fuzdb: a new phase in understanding fuzzy interactions. Nucleic Acids Res. 2022. 10.1093/nar/gkab1060. 10.1093/nar/gkab1060 PMC8728163 34791357

[69] Zeng M, Chen X, Guan D, Xu J, Wu H, Tong P, et al. Reconstituted postsynaptic density as a molecular platform for understanding synapse formation and plasticity. Cell. 2018. 10.1016/j.cell.2018.06.047. 10.1016/j.cell.2018.06.047 30078712

[70] Wang J, Zhu H, Tian R, Zhang Q, Zhang H, Hu J, et al. Physiological and pathological effects of phase separation in the central nervous system. J Mol Med (Berl). 2024. 10.1007/s00109-024-02435-7. 10.1007/s00109-024-02435-7 PMC11055734 38441598

[71] Sun J, Qu J, Zhao C, Zhang X, Liu X, Wang J, et al. Precise prediction of phase-separation key residues by machine learning. Nat Commun. 2024. 10.1038/s41467-024-46901-9. 10.1038/s41467-024-46901-9 PMC10965946 38531854

[72] Meng L, Chan W-S, Huang L, Liu L, Chen X, Zhang W, et al. Mini-review: Recent advances in post-translational modification site prediction based on deep learning. Comput Struct Biotechnol J. 2022. 10.1016/j.csbj.2022.06.045. 10.1016/j.csbj.2022.06.045 PMC9284371 35860402

[73] Buel GR, Walters KJ. Can AlphaFold2 predict the impact of missense mutations on structure? Nat Struct Mol Biol. 2022. 10.1038/s41594-021-00714-2. 10.1038/s41594-021-00714-2 PMC11218004 35046575

[74] Wayment-Steele HK, Ojoawo A, Otten R, Apitz JM, Pitsawong W, Hömberger M, et al. Predicting multiple conformations via sequence clustering and AlphaFold2. Nature. 2024. 10.1038/s41586-023-06832-9. 10.1038/s41586-023-06832-9 PMC10808063 37956700

[75] Cheng J, Novati G, Pan J, Bycroft C, Žemgulytė A, Applebaum T, et al. Accurate proteome-wide missense variant effect prediction with AlphaMissense. Science. 2023. 10.1126/science.adg7492. 10.1126/science.adg7492 37733863

[76] Chang L, Perez A. Ranking peptide binders by affinity with AlphaFold. Angew Chem Int Ed. 2023. 10.1002/anie.202213362. 10.1002/anie.202213362 36542066

[77] Wood CW, Woolfson DN. Ccbuilder 2.0: powerful and accessible coiled-coil modeling. Protein Sci. 2018. 10.1002/pro.3279. 10.1002/pro.3279 PMC5734305 28836317

[78] Wood CW, Heal JW, Thomson AR, Bartlett GJ, Ibarra AÁ, Brady RL, et al. ISAMBARD: an open-source computational environment for biomolecular analysis, modelling and design. Bioinformatics. 2017. 10.1093/bioinformatics/btx352. 10.1093/bioinformatics/btx352 PMC5870769 28582565

[79] Harmat Z, Dudola D, Gáspári Z. DIPEND: an open-source pipeline to generate ensembles of disordered segments using neighbor-dependent backbone preferences. Biomolecules. 2021. 10.3390/biom11101505. 10.3390/biom11101505 PMC8534045 34680137

[80] Offer R, Knight P. The structure of the head-tail junction of the myosin molecule. J Mol Biol. 1996. 10.1006/jmbi.1996.0096. 10.1006/jmbi.1996.0096 8604126

[81] Galvanetto N, Ivanović MT, Chowdhury A, Sottini A, Nüesch MF, Nettels D, et al. Extreme dynamics in a biomolecular condensate. 2023. Nature. 10.1038/s41586-023-06329-5 10.1038/s41586-023-06329-5 PMC11508043 37468629

[82] Hasai K. Unraveling the mysteries of dendritic spine dynamics: Five key principles shaping memory and cognition. Proc Jpn Acad Ser B Phys Biol Sci. 2023. 10.2183/pjab.99.018. 10.2183/pjab.99.018 PMC10749395 37821392

[83] Mohácsi M, Török MP, Sáray S, Tar L, Farkas G, Káli Sz. Evaluation and comparison of methods for neuronal parameter optimization using the Neuroptimus software framework. PLoS Comput Biol. 2024. 10.1371/journal.pcbi.1012039. 10.1371/journal.pcbi.1012039 PMC11706405 39715260

[84] Mosalaganti S, Obarska-Kosinska A, Siggel M, Taniguchi R, Turoňová B, Zimmerli CE, et al. Ai-based structure prediction empowers integrative structural analysis of human nuclear pores. Science. 2022. 10.1126/science.abm9506. 10.1126/science.abm9506 35679397

[85] Klukowski P, Riek R, Güntert P. Rapid protein assignments and structures from raw NMR spectra with the deep learning technique ARTINA. Nat Commun. 2022. 10.1038/s41467-022-33879-5. 10.1038/s41467-022-33879-5 PMC9579175 36257955

[86] Butler-Hallissey C, Leterrier C. Super-resolution imaging of the neuronal cytoskeleton. Curr Biol. 2023. 10.1038/s44303-024-00054-y. 10.1038/s44303-024-00054-y PMC12118673 40604119

[87] Juhász G, Madarász M, Szmola B, Fedor FZ, Balogh-Lantos Z, Szabó Á, et al. Hippocampal recording with a soft microelectrode array in a cranial window imaging scheme: a validation study. Sci Rep. 2024. 10.1038/s41598-024-75170-1. 10.1038/s41598-024-75170-1 PMC11490575 39427030

[88] de Chadarevian S. John Kendrew and myoglobin: protein structure determination in the 1950s. Protein Sci. 2018. 10.1002/pro.3417. 10.1002/pro.3417 PMC5980623 29607556

